# 5’-flanking variants of equine casein genes (*CSN1S1*, *CSN1S2*, *CSN2*, *CSN3*) and their relationship with gene expression and milk composition

**DOI:** 10.1007/s13353-018-0473-2

**Published:** 2018-10-16

**Authors:** Jakub Cieslak, Lukasz Wodas, Alicja Borowska, Piotr Pawlak, Grazyna Czyzak-Runowska, Jacek Wojtowski, Kamila Puppel, Beata Kuczynska, Mariusz Mackowski

**Affiliations:** 1 Department of Horse Breeding, Poznan University of Life Sciences, Wolynska 33, 60-637 Poznan, Poland; 2 Department of Genetics and Animal Breeding, Poznan University of Life Sciences, Wolynska 33, 60-637 Poznan, Poland; 3 Department of Animal Breeding and Product QualityAssessment, Poznan University of Life Sciences, Sloneczna 1, 62-002 Zlotniki, Poland; 4 Department of Animal Science, Cattle Breeding Division, Warsaw University of Life Sciences, Ciszewskiego 8, 02-786 Warsaw, Poland; 5 Horse Genetic Markers Laboratory, Poznan University of Life Sciences, Wolynska 33, 60-637 Poznan, Poland

**Keywords:** Equine milk, Casein genes, Polymorphism, Gene expression, Milk composition

## Abstract

Genes encoding casein proteins are important candidates for milk composition traits in mammals. In the case of the domestic horse, our knowledge of casein genes is limited mainly to coding sequence variants. This study involved screening for polymorphism in 5’-flanking regions of four genes encoding equine caseins (*CSN1S1*, *CSN1S2*, *CSN2*, and *CSN3*) and making a preliminary assessment of their effect on the gene expression (on the mRNA and protein levels) and milk composition traits in selected horse breeds. Altogether, 23 polymorphisms (21 described previously SNPs and two novel InDels) were found in the studied sequences, the majority of which are common in various horse breeds. Statistical analysis revealed that some are putatively associated with gene expression or milk composition — for example, the c.-2047_-2048insAT polymorphism (*CSN1S1*) turns out to be related to the total milk protein content in Polish Primitive Horse (*p* < 0.05), whereas c.-2105C>G SNP (*CSN2*) is related to beta-casein relative mRNA level and milk lactose concentration in the Polish Coldblood Horse breed (*p* < 0.05). We have also found significant effects of horse breed and lactation time-point on gene expression and mare’s milk composition. Our study indicates that the 5’-regulatory regions of genes encoding casein proteins are interesting targets for functional studies of their expression and the composition traits of mare’s milk.

**Electronic supplementary material:**

The online version of this article (10.1007/s13353-018-0473-2) contains supplementary material, which is available to authorized users.

## Introduction

Investigations into casein genes have been conducted for many years, as they are considered important for milk production traits. To date, the majority of studies have been conducted on ruminants (cattle, sheep, and goats) because these remain the main sources of milk consumed by humans throughout the world. Years of intense investigations have revealed the presence of numerous polymorphic variants of casein genes and proteins. Some of these have turned out to be associated with different milk composition traits and important physicochemical properties (Caroli et al. 2009). Moreover, the last decade has seen significant developments in our knowledge of the caseins of species other than ruminants. Numerous polymorphic variants of casein genes and proteins have recently been described in the llama (*Lama glama*), the camel (*Camelus dromadarius*), and the domestic horse (*Equus caballus*) (Pauciullo et al. 2014; Pauciullo and Erhardt 2015; Cieslak et al. 2016; Brinkmann et al. 2016). Of these minor dairy species, most attention has been paid to equine milk, because its composition is considered similar to human breast milk. Additionally, the multiple health-promoting features attributed to the mare’s milk make it increasingly valued as a product for human nutrition and a desirable substrate for the cosmetic industry (Salimei and Fantuz 2012). This is related predominantly with the high amounts of bioactive components (e.g., lysozyme and lactoferrin) present in mare’s milk and with its low allergenic potential (Cieslak et al. 2017). Equine milk is also increasingly recognized as a product recommended in the treatment of several human disorders (e.g., Crohn’s disease, hepatitis, gastric ulcers, cardiovascular problems) (Pieszka et al. 2016).

From the molecular point of view, casein genes are very important models of alternative splicing processes, a fact which is related to their structure (multiple short exons), and which results in frequent exon skipping events. The best-documented experiment regarding alternative splicing forms in equine caseins is that of Lenasi et al. (2006), who demonstrated that the appearance of different splicing forms of horse beta-casein (CSN2) is associated with SNPs detected in the regulatory region found in the first intron of the *CSN2* gene. Various splicing forms have also been found in the equine alpha S1 (CSN1S1) casein (Matéos et al. 2009).

Although casein concentration in mature mare’s milk is significantly lower than in cow’s milk (about 55% vs. 80% of the total milk proteins), the number of investigations into equine casein genes and proteins continues to increase. This is related to the several important roles attributed to casein proteins. The most recognizable biological function of the α_s1_ (CSN1S1), α_s2_ (CSN1S2), and β (CSN2) caseins is forming the micelles — macromolecular structures responsible for the transfer of calcium to the newborn. κ-casein (CSN3) is thought to be the factor that stabilizes the micelles. Moreover, investigations of human breast milk have indicated that CSN3 may play an important protective function against *Helicobacter pylori* infections in infants (Uniacke-Lowe et al. 2010).

Although the number of studies of polymorphic variants of equine milk proteins and their encoding genes is increasing, due to the difficulty of obtaining valuable phenotypic data, our knowledge of their effects on gene expression and mare’s milk composition traits remains very limited. In the case of equine caseins, our recently published study showed that the occurrence of *CSN1S2* polymorphic variants A and B (caused by a 1.3 kb deletion spanning two coding exons) may have an effect on the alpha-s2 casein concentration in equine milk (Cieslak et al. 2016). It is also clear that the vast majority of experiments into equine milk caseins that have been described have concentrated on gene coding regions, with the aim of finding variants that might alter the encoded protein structure. On the other hand, it is well known that polymorphisms located in the noncoding regulatory fragments may also be associated with gene expression and milk composition. This was confirmed again in recent studies of small ruminants (sheep and goats) that described the relationship between polymorphic variants located within casein gene regulatory sequences, gene expression, and variability in different milk composition traits (Noce et al. 2016; Cosenza et al. 2016).

Given the above, we performed this pilot study of four equine caseins (CSN1S1, CSN1S2, CSN2, and CSN3). The major aim of our experiment was molecular characterization of equine casein loci including screening for polymorphisms in their 5’-flanking regions and assessment of their expression levels. We also determined the potential effects of found 5’-flanking variants on both — gene expression and mare’s milk composition. Finally, we assessed the changes in equine casein gene expression (on the mRNA and milk protein levels) between various horse breeds and at three lactation time-points (postpartum weeks 5, 10, and 15).

## Material and methods

### Screening for polymorphism and genotyping

Screening for polymorphisms was performed using a genomic DNA panel consisting of 96 samples from 12 horse breeds: Polish Primitive Horse (PPH, *n* = 8), Polish Coldblood Horse (PCH, *n* = 8), Polish Warmblood Horse (PWH, *n* = 8), Arabian (ARAB, *n* = 8), Thoroughbred (THOR, *n* = 8), Welsh Pony (WELP, *n* = 8), Shetland Pony (SHET, *n* = 8), Haflinger (HAFL, *n* = 8), Percheron (PERCH, *n* = 8), Fiording (FIOR, *n* = 8), Silesian (SIL, *n* = 8), and Hucul (HUC, *n* = 8). The samples were derived from the collection of the Horse Genetic Markers Laboratory at Poznań University of Life Sciences (Poznań, Poland), where they were previously used for parentage analysis. For each gene studied, the two overlapping fragments of the 5’-flanking region were PCR amplified. The obtained amplicons harbored 1115 bp, 1036 bp, 1113 bp, and 1137 bp of the *CSN1S1*, *CSN1S2*, *CSN2*, and *CSN3* genes, respectively. PCR primers were designed using Primer3 software (Koressaar and Remm 2007) and synthesized by Sigma-Aldrich (United Kingdom). PCR amplifications were conducted in a T-300 thermocycler (BioRAD, USA) using the following conditions: initial denaturation (95 °C, 5 min), 35 cycles of denaturation (95 °C, 30 s), annealing of primers (various temperatures, 45 s), elongation (72 °C, 1 min), and final extension (72 °C, 10 min). The PCR primer sequences and other important amplification details are shown as supplementary material. PCR products length and integrity were checked by electrophoresis (120 V, 30 min) in 1.5% agarose gel stained with ethidium bromide. The screening for polymorphisms and genotyping were carried out using the Sanger sequencing method preceded by the enzymatic cleaning of PCR products (using Thermosensitive Alkaline Phosphatase and Exonuclease I) from unused primers and nucleotides. The sequencing reaction with the use of a BigDye Terminator v1.1 Cycle Sequencing Kit was performed in a T-100 thermocycler (BioRAD, USA) under the following conditions: initial denaturation (95 °C, 5 min), 25 cycles of denaturation (95 °C, 30 s), primer annealing (50 °C, 10 s), and elongation (60 °C, 4 min). After filtering through a 96-well plate with Sephadex (Sigma-Aldrich, Germany), the samples were electrophoretically separated in an ABI Prism 3130 Genetic Analyzer (Applied Biosystems, USA). The nucleotide sequences obtained in this way were then analyzed using Lasergene SeqMan Pro (version 12.2.0) software (DNAStar, USA). Afterwards, the JASPAR database resources (Khan et al. 2018) were screened to verify whether detected polymorphic variants can alter the putative consensus sequences for transcription factors.

These PCR and direct DNA sequencing techniques were also applied to genotype 74 mares used for the association analysis (the animal group is described in detail below), for which the gene expression and milk composition traits measurements were carried out.

### Gene expression and milk composition studies

All animal procedures in this study were approved by the National Commission for Ethics of Animal Experimentation, Local Ethics Committee for Animal Research (Poznań, Poland; permission number 39/2012).

Milk samples (100 ml each) were collected manually from 74 mares representing three horse breeds: PPH (*n* = 20), PWH (*n* = 27), and PCH (*n* = 27). The animals were derived from the four Polish national horse studs (Kobylniki, Sieraków, Racot, and Nowe Jankowice), where they were kept in congenial environmental conditions. The milking procedure was carried out in the morning (7–9 a.m.), providing visual contact between mothers and foals. To obtain the best characterization of changes in gene expression (and milk composition) during lactation, each mare was milked three times (in postpartum weeks 5, 10, and 15), yielding a total of 222 milk samples. The collected milk was partly (15 ml) frozen in liquid nitrogen for relative transcript level analysis, and the remaining part was stored at − 20 °C for the milk composition studies.

The total RNA was isolated from mare’s milk somatic cells using TriPure Isolation Reagent (Roche, USA), following to the methodology described in detail in our previously published article (Cieslak et al. 2016). After cDNA synthesis using a Transcriptor First Strand cDNA Synthesis Kit (Roche, USA), the relative transcript abundances of four genes encoding equine caseins (*CSN1S1*, *CSN1S2*, *CSN2*, *CSN3*) and four internal control genes (*ACTB*, *GAPDH*, *TOP2B*, and *KRT8*) were determined. The methodology of selecting the reference genes was described in our previous paper (Cieslak et al. 2015). Real-time PCR primers and TaqMan probes were designed and synthesized by TIB Molbiol, Germany. The Real-time PCR amplification was carried out in duplicate using a LightCycler 480 instrument (Roche, USA). The following cycling conditions were employed: initial denaturation (95 °C, 5 min), 45 cycles of denaturation (95 °C, 10 s), annealing of primers and probe (60 °C, 30 s), and DNA synthesis (72 °C, 1 s). The results were normalized to the geometric mean of the internal control genes’ relative mRNA levels, following the methodology described by Vandesompele et al. (2002).

The concentrations of the major components of the milk (protein, fat, and lactose) were measured using an automated infrared analysis with a Milkoscan FT2 instrument (Foss Electric, Denmark). The four milk casein protein concentrations were assessed by high-performance liquid chromatography (HPLC), using the methodology described in our recent study of equine alpha-s2 casein variants A and B (Cieslak et al. 2016). Briefly, 5 ml of solution consisting of Bis-Tris buffer (0.1 M), guanidine hydrochloride (6 M), sodium citrate (5.37 mM), and dithiothreitol (19.5 mM) was added to an equal volume of frozen milk samples. After thawing, the samples were shaken (30 s), incubated at room temperature (1 h), and centrifuged (14,000×*g*, 15 min). Afterwards, the fat layer was removed and 3 ml of the solubilized sample was diluted (1:3 proportion) with a solution containing guanidine hydrochloride and solvent A (ACN, water, and trifluoroacetic acid at 100:900:1 ratio). All samples were then filtered through a nylon filter. All four caseins concentrations were measured using an Agilent 1100 Series RP-HPLC (Agilent Technologies, Germany). The separation was carried out at ambient temperatures on a Jupiter column 5μ C18 300A (Phenomenex, USA). The peaks obtained in this manner were compared with proper bovine casein protein standards (Sigma-Aldrich, Germany).

### Statistical methods

The restricted maximum likelihood (REML) method was used to estimate the unknown variance components. Initially, the importance of each factor included in the statistical model was examined using the Kruskall–Wallis and Friedman tests. A model encompassing the fixed effect of breed and sampling time (postpartum weeks 5, 10, or 15) as a repeated-measure factor was used in order to verify the potential effect of the casein gene polymorphisms on their relative transcript abundances (measured in milk somatic cells), the milk’s content of particular caseins, and the milk’s composition (as concentrations of protein, fat, and lactose). Hypotheses were tested with the *F* test and the multiple comparison procedure based on least significant differences (LSDs), followed by the Tukey–Kramer adjustment. Genotype groups containing less than five horses (15 measurements) were excluded from the association study. All the statistical analysis was carried out using the SAS 9.3 package (SAS Institute, USA).

## Results

### Casein gene 5’-flanking variants

In total, 23 polymorphic sites, 21 SNPs (present already in European Variants Archive database) and two novel InDels, were detected in the 5’-flanking regions of the four equine casein genes. Over one third of these sites (8) were found within the *CSN3* gene sequence. The majority of these kappa-casein gene variants were widely distributed across the horse breeds (Table 1). On the other hand, only three genetic variants were found within the corresponding region of the *CSN1S2* gene. It should also be noted that, except for the c.-1732A>G polymorphism of the *CSN1S1* gene (detected only in the HUC and SIL breeds), all the detected variants were present in at least one of the three breeds (PPH, PWH, or PCH) for which the gene expression and milk composition analyses were carried out. However, many of these ultimately had to be removed from the association studies on account of their very low frequencies.

**Table 1:** Polymorphisms detected in 5’-flanking regions of equine casein genes

Gene	Polymorphism (EVA accession number)	Breed
*CSN1S1*	c.-1665C>T (rs1143347375)	ARAB, THOR, PCH, PWH, WELP
	c.-1667T>C (rs1146618007)	FIOR, PCH, PERCH, PWH, SHET, WELP
	c.-1732A>G (rs396970024)	HUC, SIL
	c.-1917G>T (rs1144190448)	FIOR, PCH, PERCH, PWH, SHET, WELP
	c.-1918T>A (rs1143280124)	FIOR, PCH, PPH, SHET, THOR
	c.-1922C>G (rs1139127764)	PCH, PWH
	c.-2168G>C (rs1138196331)	PCH, PWH, THOR, WELP
*CSN1S2*	c.-2047_-2048insAT (novel)	ARAB, FIOR, HAFL, PCH, PPH, PWH, SHET, WELP
	c.2121T>C (rs1141063741)	ARAB, HUC, PCH, PWH, SHET, THOR
	c.-2543G>A (rs1140158443)	HAFL, HUC, PPH, WELP
*CSN2*	c.-2105C>G (rs1144253835)	ARAB, HAFL, PCH, PPH, PWH, SIL, THOR
	c.-2429C>T (rs1138717818)	ARAB, HAFL, PCH, SIL
	c.-2763_-2764delTT (novel)	ARAB, HUC, PCH, PERCH, PPH, PWH
	c.-2817T>C (rs1143881137)	PCH, PWH, SHET, SIL, WELP
	c.-2973C>G (rs1145121702)	HUC, PCH, PPH, PWH, SHET
*CSN3*	c.-2756A>G (rs1143034651)	FIOR, HUC, PCH, PERCH, PPH, PWH, SHET, SIL, THOR, WELP
	c.-2925C>G (rs1143951555)	FIOR, HUC, PCH, PERCH, PPH, PWH, SHET, SIL, THOR, WELP
	c.-2970C>T (rs1140546235)	FIOR, HAFL, HUC, PCH, PPH, PWH, SHET, WELP
	c.-3012G>C (rs1141704454)	ARAB, FIOR, HAFL, HUC, PCH, PERCH, PPH, PWH, SHET, SIL, THOR, WELP
	c.-3109A>G (rs1152193036)	FIOR, HAFL, HUC, PCH, PERCH, PPH, PWH, SHET, SIL, WELP
	c.-3515G>A (rs1139287681)	ARAB, HAFL, HUC, PPH, PWH, SIL
	c.-3669G>C (rs1142437750)	FIOR, HUC, PCH, PPH, PWH, PERCH, SHET, SIL, WELP
	c.-3711 T>C (rs1151652741)	FIOR, HUC, PCH, PERCH, PPH, SHET, SIL, WELP

*EVA* European Variation Archive (https://www.ebi.ac.uk/eva/)

**Fig. 1:**
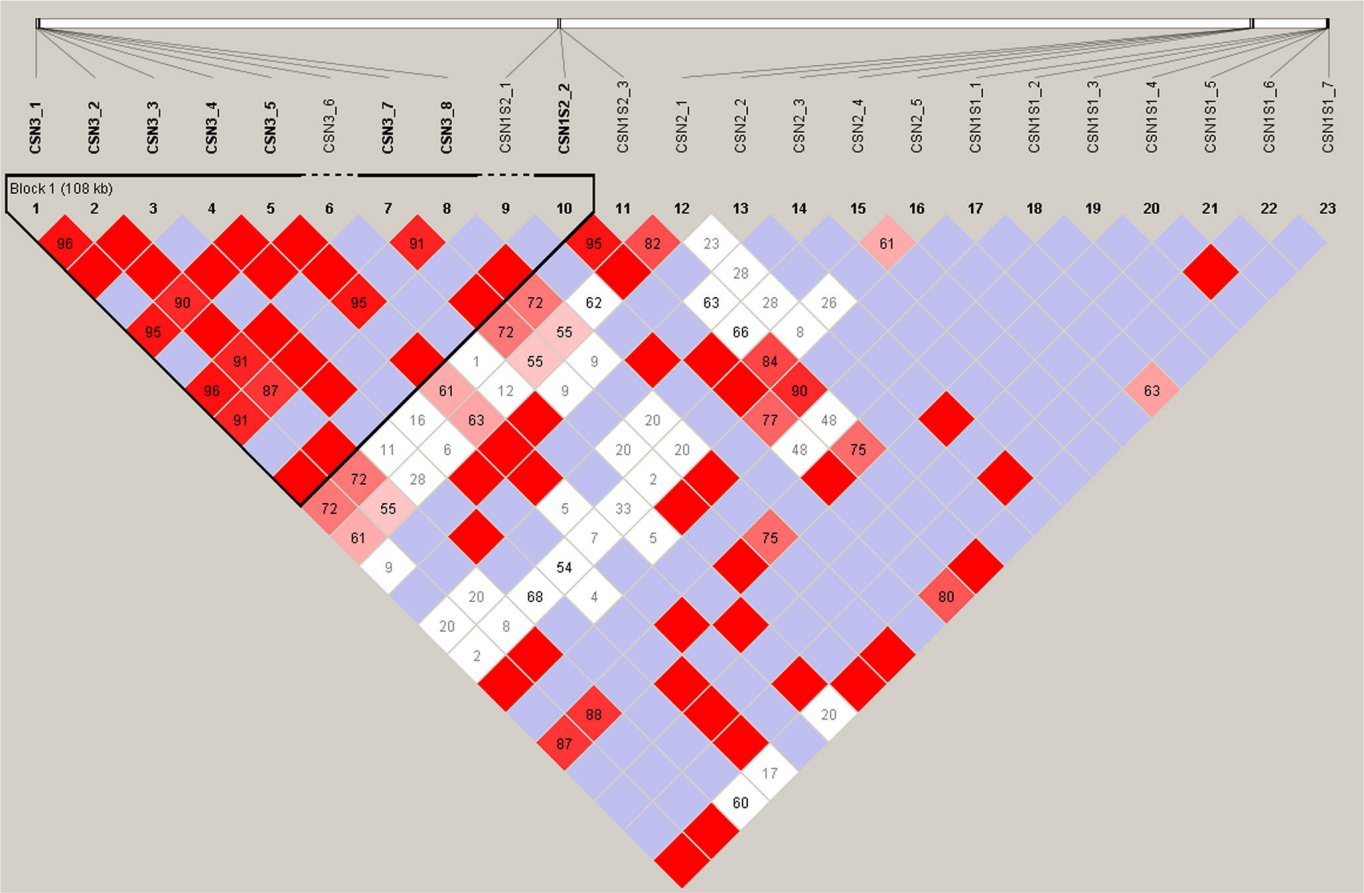
LD across analyzed fragment of ECA3 (HaploView software). Numbers in blocks denote D’ values. Red blocks without numbers show full LD between pairs of loci. Blue blocks without numbers denote a lack of LD between pairs of loci

As in other mammalian species, all the genes encoding the four equine caseins share the same (~ 300 kb) chromosomal region (ECA3). We have therefore checked for potential linkage disequilibrium (LD) between the investigated SNPs using HaploView software (Barrett et al. 2005). The LD values between the analyzed pairs of loci varied from almost no LD to full LD. Regions of high LD score were unevenly distributed across the studied fragment of chromosome 3. The analysis indicated the presence of one clear haplotype block spanning almost all (7/8) *CSN3* and one of the three *CSN1S2* polymorphisms. However, in several cases, the full LD was detected even between variants located in the most distant genes (*CSN3* and *CSN1S1*) (Fig. 1).

Analysis with the application of JASPAR database resources has revealed that 17 of 22 detected polymorphic sites can alter the predicted binding sites for transcription factors (see supplementary material). It justifies the necessity of their further testing within the term of potential impact on gene expression and milk composition.

### Casein gene expression on the transcript and protein levels

Comparison of the particular casein protein concentrations in the milk of three horse breeds (PPH, PWH, PCH) revealed the highest amount of alpha-caseins (CSN1S1 and CSN1S2) in PCH, while the CSN2 and CSN3 proteins were most abundant in the PPH breed (Table 2). These results are not fully in agreement with the relative transcript abundances of each gene measured in the milk somatic cells. For example, the highest relative mRNA abundance of the *CSN1S1*, *CSN2*, and *CSN3* genes was noticed for PWH while, in the case of *CSN1S2*, the most elevated gene expression level was observed for the PPH breed.

**Table 2:** Interbreed comparison of four equine casein transcript levels and milk protein concentrations (all lactation time-points together). Presented values are means and standard errors (SEM)

Breed	*CSN1S1*		*CSN1S2*		*CSN2*		*CSN3*	
	mRNA (RA)	Protein (g/L)	mRNA (RA)	Protein (g/L)	mRNA (RA)	Protein (g/L)	mRNA (RA)	Protein (g/L)
PPH	474.51^A^ (44.80)	3.49^a^ (0.11)	247.60^Aa^ (28.67)	0.55 (0.04)	1039.51^A^ (114.81)	1.58 (0.08)	38.84^a^ (5.37)	0.72^A^ (0.03)
PWH	598.82^A^ (57.49)	3.72^ab^ (0.11)	128.37^B^ (22.47)	0.49 (0.03)	1577.11^B^ (129.03)	1.41 (0.07)	48.01^Aa^ (9.27)	0.67^A^ (0.03)
PCH	231.74^B^ (23.71)	3.93^b^ (0.10)	171.44^b^ (25.74)	0.60 (0.04)	761.62^A^ (59.83)	1.49 (0.08)	17.64^Bb^ (1.94)	0.55^B^ (0.02)

*RA* relative abundance, *PPH* Polish Primitive Horse, *PWH* Polish Warmblood Horse, *PCH* Polish Coldblood Horse. Values marked with different superscripts differed significantly at *p* < 0.01(uppercase letters) or *p* < 0.05 (lowercase letters)

Similar comparisons carried out for the three different lactation time-points (postpartum weeks 5, 10, and 15) showed various tendencies for each casein concentrations. The amount of CSN1S1 in milk increased significantly (*p* < 0.01) between weeks 5 and 10 and remained stable until the last time-point (week 15). The concentration of the CSN2 protein was almost constant over the whole experiment range, whereas the concentration of CSN3 showed a significant decrease between weeks 5 and 15 (*p* < 0.05) (Table 3). Interestingly, the relative mRNA abundance of each casein gene showed a similar time-dependent pattern: a decrease in expression between weeks 5 and 10, followed by an increase of relative transcript level measured at week 15.

**Table 3:** Comparison of four equine casein transcript levels and milk protein concentrations between the three lactation time-points (all breeds together). Presented values are means and standard errors (SEM)

Lactation period (week postpartum)	*CSN1S1*		*CSN2*		*CSN3*		*CSN1S2*	
	mRNA (RA)	Protein (g/L)	mRNA (RA)	Protein (g/L)	mRNA (RA)	Protein (g/L)	mRNA (RA)	Protein (g/L)
5	554.79^A^ (68.89)	3.42^A^ (0.09)	1168.00^AB^ (142.61)	1.49 (0.06)	58.96^A^ (10.87)	0.71^a^ (0.03)	Data not shown^*^	
10	313.82^B^ (28.88)	3.89^B^ (0.11)	913.56^A^ (70.67)	1.48 (0.07)	22.26^B^ (2.99)	0.64^ab^ (0.02)		
15	441.25^AB^ (36.89)	3.88^B^ (0.11)	1346.30^B^ (113.76)	1.49 (0.09)	23.66^B^ (2.08)	0.59^b^ (0.02)		

*Results previously presented (Cieslak et al. 2016). *RA* relative abundance. Values marked with different superscripts differed significantly at *p* < 0.01 (uppercase letters) or *p* < 0.05 (lowercase letters)

### Relationship between selected polymorphisms, gene expression, and milk composition

The association study of those polymorphisms that occurred with sufficient frequency within each horse breed (at least five individuals in each genotypic group) revealed several statistically significant relationships. In the case of the *CSN1S2* gene c.-2047_-2048insAT polymorphism, the PPH individuals carrying the *del/del* genotype showed lower mean total milk protein content (1.66 g/100 mL) than did the *ins/ins* and *ins/del* genotypes (1.83 g/100 mL), *p* < 0.05. The heterozygous *GC* genotype in the c.-2105C>G SNP of the *CSN2* gene was associated with an increased mean beta-casein relative transcript level and lower milk lactose content, compared to the *CC* homozygotes in the PCH breed (6.41 and 6.51 g/100 mL, respectively, *p* < 0.05). Finally, within the PPH breed, the c.-3669G>C *GG* genotype carriers (and the *TT* homozygous individuals in the c.-3711T>C SNP located within the *CNS3* gene — both polymorphisms are in a strong LD) show a significantly (*p* < 0.05) elevated mean kappa-casein (0.79 g/L) and total milk protein content (1.77 g/100 mL), as compared with horses bearing other genotypes. Moreover, an increased CSN3 protein content was noted for the CC genotype of c.-2925C>G SNP (*p* < 0.05). No significant relationship with gene expression or milk composition traits was recorded for the *CSN1S1* gene 5’-flanking variants. The detailed results of the association studies are presented as supplementary material.

## Discussion

Although equids (horses and donkeys) are currently considered minor dairy species, research indicates that the consumption of equine milk is one of the oldest forms of horse utility (Outram et al. 2009). With the current broad interest in the production and utility of functional food, numerous health-promoting properties traditionally attributed to equine milk are being revisited, and the number of investigations into its composition is constantly increasing (Pieszka et al. 2016).

Previous studies have shown clearly that, like other species, the concentration of particular milk components (including casein proteins) in equine milk is variable, even within the same horse breed (Cieslak et al. 2016). Taking into account the values of heritability (*h*^2^) estimated for particular casein amounts in cows’ milk (Bonfatti et al. 2011), we can assume that a significant fraction of the variation in milk casein quantities in equine milk is due to genetic factors. To date, however, the majority of studies have focused on detecting polymorphisms and analyzing their distribution in various horse breeds (Brinkmann et al. 2016; Hobor et al. 2008; Selvaggi et al. 2010). Knowledge of the potential functionality of these genetic variants remains very limited. Furthermore, although the existence of polymorphic forms of equine casein gene coding sequence has generally been well described, recent studies involving ruminants have confirmed that some important variants affecting casein expression can also be located in the regulatory sequences of the encoding genes (Noce et al. 2016; Cosenza et al. 2016). For this reason, we decided to conduct this preliminary study concentrated on the 5’-flanking regions of four equine casein genes.

The level of polymorphism of the studied sequences varied between ~ 0.2 polymorphic site per 100 bp (*CSN1S2*) and ~ 0.7 polymorphic site per 100 bp (*CSN3*). The vast majority of the genetic variants were present in a large number of horse breeds, which may suggest that these polymorphisms are phylogenetically old. Interestingly, many of these variants were present even in small indigenous horse breeds that originated from a very limited number of founders, such as the PPH and the HUC breeds (14 and 13 polymorphisms, respectively). A similar high level of casein gene coding sequence variation was described recently for the Icelandic Horse breed, which is also considered to be an old, indigenous horse breed with a closed registry (no outside blood is accepted) (Brinkmann et al. 2016). Unfortunately, many of genetic variants we detected had very low frequencies in the three horse breeds for which milk composition traits were available (PPH, PCH, and PWH), and ultimately had to be excluded from the association study. Since it is well known that rare variants can also significantly contribute to the variability of quantitative traits (Zhang et al. 2016), a greater population of horses would be needed to assess the potential effects of all the detected 5’-flanking variants on equine milk composition. On the other hand, it should be underlined that our project included over 70 horses and 222 milk samples, which make it one of the largest studies of equine milk composition to have been conducted. This fact is due to the difficulties in collecting the appropriate number of samples from horses of the same breed, being kept in similar environmental conditions — which would seem to be crucial if reliable studies of the genetic background of milk composition variability are to be conducted. Moreover, even on commercial equine dairy farms, milk production and composition traits are not routinely recorded, and such phenotypes must be collected by researchers *de novo* (Brinkmann et al. 2016).

Our preliminary study of the potential relationship between the detected polymorphic variants, casein gene expression, and basic milk composition traits revealed several statistically significant associations for all the investigated genes, except for *CSN1S1*. This suggests that — similarly to experiments involving ruminants — genes encoding casein proteins should be extensively studied as promising candidates for equine milk composition. Moreover, the analysis of the LD structure between particular SNPs located in the casein cluster on ECA3 has clearly shown that, if a larger genotyped and phenotyped animal group were available, association studies of particular haplotypes could be carried out analogically to investigations conducted on ruminant casein genes (Nilsen et al. 2009; Hayes et al. 2006).

As in our previous reports, we have noticed a significant effect of horse breed and lactation time-point on gene expression and milk composition (concentration of particular casein proteins). However, the observed profiles of gene expression measured at the relative mRNA stage were different from the results assessed at protein level. These results are in agreement with the position of many earlier papers, indicating there is no linear relationship between mRNA abundance given the protein synthesis rate. This mainly due to the diversity of translation efficiency and the numerous mechanisms that affect gene expression processes (Maier et al. 2009). We can thus conclude that the results of each experiment regarding differences in gene expression should be interpreted very carefully, especially where the assessment of final protein level was not performed.

Our study indicates that the 5’-regulatory regions of genes encoding casein proteins are interesting targets for functional studies of their expression and of mare’s milk composition traits. However, further analyses involving larger groups of animal and additional methods (such as molecular analysis of predicted transcription factor binding sites) are needed to fully characterize the functionality of equine casein gene 5’-flanking regions.

### Statement of author contributions

JC: Experimental design, animal genotyping, results data analysis, manuscript preparation

MM and LW: Molecular technique optimization, animal genotyping, manuscript revision

PP: Gene expression studies, manuscript revision

GC-R and JW: Basic milk composition analyses, manuscript revision

KP and BK: Milk casein fraction analyses, manuscript revision

AB: Statistical analysis, manuscript revision

## Electronic supplementary material

ESM 1 (PDF 115 kb)

ESM 2 (PDF 430 kb)

ESM 3 (PDF 401 kb)
