# Supplementary material for: 5’-flanking variants of equine casein genes (CSN1S1, CSN1S2, CSN2, CSN3) and their relationship with gene expression and milk composition
Source: J Appl Genet. 2018 Oct 16;60(1):71–8. doi: 10.1007/s13353-018-0473-2 (PMC6373402; doi:10.1007/s13353-018-0473-2)
Supplement: Supplementary file 1 — (PDF 115 kb) [file 13353_2018_473_MOESM1_ESM.pdf]

Supplementary material 1. PCR Primer sequences and amplification details.

Primers (probes)<br>Sequences	PCR product size (bp)	Annealing temp. (°C)	No. of cycles	Polymerase
**Screening for polymorphisms and genotyping**				
CSN1S1_IF: ATGGTGCCCAAAACAATTA<br>CSN1S1_IR: TTGAACCCAAGATTGGGAAG	661	60	35	DreamTaq (Thermo Scientific)
CSN1S1_IIF: GAATGTTGTGGCTGGTTTGA<br>CSN1S1_IIR: TTTCCAACATCATTGGCTCA	639	62	35	Perpetual Taq (EurX)
CSN1S2_IF: GAGAGCCGAGTTTCCCATTA<br>CSN1S2_IR: TGAAAACATTACCCTGTTTCCT	563	60	35	DreamTaq (Thermo Scientific)
CSN1S2_IIF: TGCACTTGAGCAGAATCCAG<br>CSN1S2_IIR: TTGCTTATAGTCTGCCAGCATT	550	60	35	DreamTaq (Thermo Scientific)
CSN2_IF: AATGGCCCTGGGAACTAAG<br>CSN2_IR: TTTCCAAGGTGGAGGACAAG	549	60	35	DreamTaq (Thermo Scientific)
CSN2_IIF: CAACACCAATACCCCAGTCC<br>CSN2_IIR: CCGCATTTTCATGCTAACCT	686	60	35	Taq (EurX)
CSN3_IF: ATGGCCCCTTTCTTTATGCT<br>CSN3_IR: TTTGCAGGTCAGGTCTTGCT	616	60	35	Taq (EurX)
CSN3_IIF: GCTTTCTAGGGTTCTGAACATTG<br>CSN3_IIR: CTTCCTTCCAGAATCGCAAC	654	60	35	Taq (EurX)
**Gene expression studies**				
ACTBF: TCCTTCCTGGGGCATGGAATC<br>ACTBR: TCCTGTCGGCGATGCCT<br>ACTBprobe: CCGTAAGGACCTGTACGCCAACACAGT	145	60	45	LightCycler 480 Probes Master (Roche)
GAPDHF: GAGGACCAGGTTGTCTCCTGC<br>GAPDHR: ATGAGCTTGACAAAGTGGTCGTT<br>GAPDHprobe: ACCCACTCTTCCACCTTCGATGCT	101	60	45	
TOP2BF: GCCAGCTGACAATAAACAGAGG<br>TOP2BR: TGCCTTTCCCATTATTCCAA<br>TOP2Bprobe: TTGATCCTGAATCTAACATTATAAGCA	101	60	45	
KRT8F: ACCCAGGAGAAGGAGCAGAT<br>KRT8R: GCTCCACTTGGTCTCCAGAA<br>KRT8probe: GCATCTGGAACAGCAGAACA	108	60	45	
CSN1S1F: AGACTGAGCAAATACAACCAACTCC<br>CSN1S1R: CTCATAGGCTTTCTCTGGCTGTTT<br>CSN1S1probe: CCATGCCCAGGAACAACTTATCAGAATGAA	95	60	45	
CSN1S2F: CACCACCTAAAACAACTGAACAAAAT<br>CSN1S2R: AGTCCCCTGTCTTAGTCTGATCCC<br>CSN1S2probe: CAACCTCGGATTGTCCTGACCCC	127	60	45	
CSN2F: CAGCAAAGAGAGGTTGAACGC<br>CSN2R: CAGGATGCTTTGTGGAACGAC<br>CSN2probe: TGTCCAGCCACAGCCTGTAGTCTATCCT	114	60	45	
CSN3F: AATATCTACCCATCCACTGTGGTACG<br>CSN3R: GTTTCAGCAAGACCTTTGTTGTC<br>CSN3probe: ACCAACAGTGAACAATGCAGTCATTCCAGA	296	60	45	

Cieslak et. al. (2018) 5’-flanking variants of equine casein genes (CSN1S1, CSN1S2, CSN2, CSN3) and their relationship with gene expression and milk composition. *Journal of Applied Genetics.*
