# Supplementary material for: 5’-flanking variants of equine casein genes (CSN1S1, CSN1S2, CSN2, CSN3) and their relationship with gene expression and milk composition
Source: J Appl Genet. 2018 Oct 16;60(1):71–8. doi: 10.1007/s13353-018-0473-2 (PMC6373402; doi:10.1007/s13353-018-0473-2)
Supplement: Supplementary file 2 — (PDF 430 kb) [file 13353_2018_473_MOESM2_ESM.pdf]

Supplementary material 2. Impact of detected polymorphic sites on consensus sequences for transcription factors

Polymorphism	***Predicted TF binding sites****	
	CSN1S1	
c.-1665C>T	**Allele C**	**Allele T**
	-	FOS, JUN, JDP2
c.-1732A>G	**Allele A**	**Allele G**
	RHOXF1	RHOXF1, ETS1, NFATC1, NFATC2, NFATC3
c.-1917G>T	**Allele G**	**Allele T**
	NFIC, GATA1, GATA2	NFIC, PRRX2, DLX3, DLX4, DLX2
c.-1922C>G	**Allele C**	**Allele G**
	GATA2, RHOXF1, NFIC, NFIX, NFYB	GATA2, CEBPB, JUN, CEBPA, FOS::JUN
c.-2168G>C	**Allele G**	**Allele C**
	GATA1, GATA2, MZF1, NFKB1	GATA1, GATA2, HIC2
	CSN1S2	
c.-2047_2048insAT	**Allele + (ins)**	**Allele - (del)**
	NOTO, Nkx2-5, Arid3a, Barhl1, NKX6-2	NOTO, Nkx2-5, Pdx1
c.-2543G>A	**Allele G**	**Allele A**
	NR4A2, Mafb	NR4A2, SOX10
	CSN2	
c.-2105C>G	**Allele C**	**Allele G**
	RUNX3, RUNX1, RUNX2, ZNF354C, LIN54	MEIS1
c.-2429C>T	**Allele C**	**Allele T**
	-	Rxra, NR4A2
c.-2817T>C	**Allele T**	**Allele C**
	FOXC1, MEIS1	FOXC1
c.-2973C>G	**Allele C**	**Allele G**
	Bhlha15, HLTF, FOXL1	Bhlha15, HLTF, FOXL1, MEIS1
	CSN3	
c.-2925C>G	**Allele C**	**Allele G**
	MZF1, ETS1	-
c.-2970C>T	**Allele C**	**Allele T**
	Ahr::Arnt	TBX21, MGA, TBX1, TBR1, EOMES
c.-3012G>C	**Allele G**	**Allele C**
	GATA2, GATA3, FOXL1, HOXA5	GATA2, GATA3, FOXL1
c.-3515G>A	**Allele G**	**Allele A**
	RHOXF1	-
c.-3669G>C	**Allele G**	**Allele C**
	-	BARHL2
c.-3711T>C	**Allele T**	**Allele C**
	FOXC1, SPIB, ETS1, ARID3A,	FOXC1, NR2E1

TF – transcription factors, *only 1-5 consensus sites with the highest relative scores are shown (according to JASPAR database)

Cieslak et. al. (2018) 5'-flanking variants of equine casein genes (CSN1S1, CSN1S2, CSN2, CSN3) and their relationship with gene expression and milk composition. *Journal of Applied Genetics.*
