# Supplementary material for: 5’-flanking variants of equine casein genes (CSN1S1, CSN1S2, CSN2, CSN3) and their relationship with gene expression and milk composition
Source: J Appl Genet. 2018 Oct 16;60(1):71–8. doi: 10.1007/s13353-018-0473-2 (PMC6373402; doi:10.1007/s13353-018-0473-2)
Supplement: Supplementary file 3 — (PDF 401 kb) [file 13353_2018_473_MOESM3_ESM.pdf]

Supplementary material 3. Association of selected equine casein 5'-flanking variants, gene expression and basic milk composition.

Table 1. Alpha-s1 casein (CSN1S1).

<table>
<tr><th rowspan="3">SNP</th><th rowspan="3">Genotype</th><th colspan="6">CSN1S1</th><th colspan="9">Basic milk components</th></tr>
<tr><th colspan="3">mRNA (RA)</th><th colspan="3">Milk protein concentration (g/L)</th><th colspan="3">Protein (g/100mL)</th><th colspan="3">Fat (g/100mL)</th><th colspan="3">Lactose (g/100mL)</th></tr>
<tr><th>PCH</th><th>PWH</th><th>PPH</th><th>PCH</th><th>PWH</th><th>PPH</th><th>PCH</th><th>PWH</th><th>PPH</th><th>PCH</th><th>PWH</th><th>PPH</th><th>PCH</th><th>PWH</th><th>PPH</th></tr>
<tr><td rowspan="3">**c.-1918T>A**</td><td>**AA**</td><td rowspan="7">n/a</td><td rowspan="3">n/a</td><td>521.68 (144.98)</td><td rowspan="7">n/a</td><td rowspan="3">n/a</td><td>3.37 (0.33)</td><td rowspan="7">n/a</td><td rowspan="3">n/a</td><td>1.59 (0.07)</td><td rowspan="7">n/a</td><td rowspan="5">n/a</td><td>1.97 (0.23)</td><td rowspan="7">n/a</td><td rowspan="5">n/a</td><td>6.56 (0.08)</td></tr>
<tr><td>**TA**</td><td>462.37 (83.70)</td><td>3.28 (0.19)</td><td>1.70 (0.04)</td><td>1.86 (0.13)</td><td>6.46 (0.04)</td></tr>
<tr><td>**TT**</td><td>472.91 (60.05)</td><td>3.62 (0.13)</td><td>1.73 (0.02)</td><td>1.70 (0.09)</td><td>6.42 (0.03)</td></tr>
<tr><td rowspan="2">**c.-1665C>T**</td><td>**CC**</td><td>639.12 (56.89)</td><td rowspan="4">n/a</td><td>3.76 (0.11)</td><td rowspan="4">n/a</td><td>1.79 (0.02)</td><td rowspan="4">n/a</td><td rowspan="4">n/a</td><td rowspan="4">n/a</td></tr>
<tr><td>**TC**</td><td>481.30 (95.35)</td><td>3.66 (0.19)</td><td>1.80 (0.03)</td></tr>
<tr><td rowspan="2">**c.-2168G>C**</td><td>**CG**</td><td>648.43 (104.14)</td><td>3.9225 (0.21)</td><td>1.77 (0.03)</td><td>1.43 (0.11)</td><td>6.54 (0.05)</td></tr>
<tr><td>**GG**</td><td>582.94 (56.12)</td><td>3.68 (0.11)</td><td>1.80 (0.02)</td><td>1.37 (0.06)</td><td>6.45 (0.03)</td></tr>
</table>

n/a – not analyzed due to low frequency of given polymorphism

Table 2. Alpha-s2 casein (CSN1S2). Values marked with different lowercase letters differed significantly (p<0.05).

SNP	Genotype	CSN1S2						Basic milk components								
		mRNA (RA)			Milk protein concentration (g/L)			Protein (g/100mL)			Fat (g/100mL)			Lactose (g/100mL)		
		PCH	PWH	PPH	PCH	PWH	PPH	PCH	PWH	PPH	PCH	PWH	PPH	PCH	PWH	PPH
c.-2047_-2048insAT	ins/ins		133.69 (29.84)	206.63 (169.77)		0.53 (0.05)	0.40 (0.22)		1.80 (0.02)	1.83[a] (0.09)		1.62 (0.22)	1.58 (0.33)		6.45 (0.04)	6.38 (0.12)
	ins/del		173.70 (31.17)	249.01 (75.92)		0.55 (0.05)	0.62 (0.09)		1.81 (0.02)	1.83[a] (0.04)	n/a	1.66 (0.16)	1.82 (0.15)	n/a	6.50 (0.04)	6.42 (0.05)
	del/del	n/a	232.66 (54.05)	237.11 (45.94)	n/a	0.56 (0.08)	0.54 (0.05)	n/a	1.72 (0.04)	1.66[b] (0.02)		1.577 (0.07)	1.78 (0.09)		6.45 (0.07)	6.46 (0.03)
c.-2121T>C	CC		211.75 (54.69)			0.67 (0.08)			1.80 (0.04)			1.41 (0.14)			6.52 (0.07)	
	TC		159.57 (27.95)	n/a		0.52 (0.04)	n/a		1.79 (0.02)	n/a	n/a	1.34 (0.07)	n/a	n/a	6.49 (0.03)	n/a
	TT		150.84 (34.87)			0.53 (0.05)			1.80 (0.02)			1.42 (0.09)			6.43 (0.04)	

n/a – not analyzed due to low frequency of given polymorphism

Table 3. Beta casein (CSN2). Values marked with different lowercase letters differed significantly (p<0.05).

SNP	Genotype	CSN2						Basic milk components								
		mRNA (RA)			Milk protein concentration (g/L)			Protein (g/100mL)			Fat (g/100mL)			Lactose (g/100mL)		
		PCH	PWH	PPH	PCH	PWH	PPH	PCH	PWH	PPH	PCH	PWH	PPH	PCH	PWH	PPH
**c.-2105C>G**	CC	**1468.14[a] (129.66)**	733.46 (81.47)	1079.25 (133.51)	1.54 (0.11)	1.43 (0.08)	1.67 (0.09)	1.65 (0.03)	1.80 (0.02)	1.72 (0.02)	1.52 (0.08)	1.41 (0.05)	1.84 (0.09)	**6.51[a] (0.02)**	6.48 (0.02)	6.45 (0.03)
	GC	**2103.93[b] (281.78)**	779.83 (79.41)	920.46 (210.06)	1.45 (0.11)	1.37 (0.18)	1.41 (0.15)	1.65 (0.03)	1.77 (0.03)	1.68 (0.041)	1.65 (0.08)	1.22 (0.12)	1.66 (0.13)	**6.41[b] (0.02)**	6.45 (0.06)	6.43 (0.04)
**c.-2429C>T**	CC	773.73 (70.23)			1.58 (0.09)			1.67 (0.03)			1.61 (0.07)			6.48 (0.02)		
	CT	754.90 (103.58)		n/a	1.37 (0.14)		n/a	1.62 (0.04)		n/a	1.57 (0.11)	n/a	n/a	6.46 (0.03)	n/a	n/a
	TT	500.46 (286.63)	n/a		1.03 (0.40)	n/a		1.47 (0.12)	n/a		1.47 (0.31)			6.25 (0.10)		
**c.-2973C>G**	CC	659.14 (91.28)		931.90 (128.12)	1.47 (0.13)		**1.50[a] (0.09)**	1.64 (0.04)		1.70 (0.02)			1.77 (0.08)			
	CG	847.49 (83.08)		1331.52 (219.35)	1.51 (0.12)		**1.82[b] (0.16)**	1.65 (0.03)		1.75 (0.04)	n/a	n/a	1.83 (0.15)	n/a	n/a	n/a
	GG	731.52 (142.31)		n/a	1.54 (0.21)		n/a	1.65 (0.06)		n/a			n/a			

n/a – not analyzed due to low frequency of given polymorphism

Table 4. Kappa-casein (CSN3). Values marked with different lowercase letters differed significantly (p<0.05).

SNP	Genotype	CSN3						Basic milk components								
		mRNA (RA)			Milk protein concentration (g/L)			Protein (g/100mL)			Fat (g/100mL)			Lactose (g/100mL)		
		PCH	PWH	PPH	PCH	PWH	PPH	PCH	PWH	PPH	PCH	PWH	PPH	PCH	PWH	PPH
**c.-3711T>C**	CC	12.19 (5.08)	n/a	27.81 (15.21)	0.51 (0.05)	n/a	**0.55[a] (0.09)**	1.54 (0.06)	n/a	**1.59[a] (0.06)**	1.47 (0.16)	n/a	1.97 (0.24)	6.50 (0.05)	n/a	6.56 (0.08)
	TC	15.54 (2.70)		43.69 (8.78)	0.56 (0.02)		**0.65[a] (0.05)**	1.67 (0.03)		**1.63[a] (0.03)**	1.63 (0.08)		1.73 (0.13)	6.46 (0.03)		6.44 (0.05)
	TT	22.39 (3.30)		39.01 (6.30)	0.57 (0.03)		**0.79[b] (0.03)**	1.66 (0.04)		**1.77[b] (0.02)**	1.57 (0.10)		1.78 (0.09)	6.46 (0.03)		6.43 (0.03)
**c.-3669G>C**	CC	12.23 (4.54)	n/a	27.81 (15.21)	0.49 (0.04)	n/a	**0.55[a] (0.09)**	1.58 (0.05)	n/a	**1.59[a] (0.06)**	1.44 (0.14)	n/a	1.97 (0.24)	6.49 (0.04)	n/a	6.56 (0.08)
	GC	16.49 (2.95)		43.69 (8.78)	0.56 (0.02)		**0.65[a] (0.05)**	1.66 (0.03)		**1.63[a] (0.03)**	1.66 (0.09)		1.73 (0.13)	6.45 (0.03)		6.43 (0.04)
	GG	21.74 (3.15)		39.01 (6.30)	0.58 (0.03)		**0.79[b] (0.03)**	1.67 (0.04)		**1.77[b] (0.02)**	1.58 (0.09)		1.78 (0.09)	6.47 (0.03)		6.43 (0.03)
**c.-3515G>A**	GA	n/a	32.64 (20.90)	36.32 (8.10)	n/a	0.68 (0.07)	0.80 (0.05)	n/a	1.86 (0.04)	1.76 (0.04)	n/a	1.17 (0.13)	1.60 (0.12)	n/a	6.53 (0.07)	6.43 (0.05)
	GG		51.01 (9.62)	40.95 (6.02)		0.67 (0.03)	0.68 (0.03)		1.78 (0.02)	1.68 (0.03)		1.42 (0.06)	1.89 (0.09)		6.47 (0.03)	6.46 (0.03)
**c.-2756A>G**	AA	22.3948 (3.3067)	n/a	43.55 (6.27)	0.57 (0.03)	n/a	0.76 (0.03)	1.66 (0.04)	n/a	1.75 (0.03)	1.58 (0.11)	n/a	1.75 (0.09)	6.46 (0.04)	n/a	6.42 (0.03)
	AG	15.5465 (2.7013)		34.87 (8.74)	0.56 (0.02)		0.72 (0.05)	1.67 (0.03)		1.68 (0.04)	1.64 (0.09)		1.79 (0.14)	6.46 (0.03)		6.47 (0.05)
	GG	12.1984 (5.0874)		27.81 (15.14)	0.515 (0.05)		0.55 (0.09)	1.55 (0.06)		1.59 (0.07)	1.47 (0.16)		1.97 (0.24)	6.50 (0.06)		6.56 (0.08)

Table 4 (continuation). Kappa-casein (CSN3). Values marked with different lowercase letters differed significantly (p<0.05).

SNP	Genotype	CSN3						Basic milk components								
		mRNA (RA)			Milk protein concentration (g/L)			Protein (g/100mL)			Fat (g/100mL)			Lactose (g/100mL)		
		PCH	PWH	PPH	PCH	PWH	PPH	PCH	PWH	PPH	PCH	PWH	PPH	PCH	PWH	PPH
c.-2925C>G	CC	22.39 (3.30)	n/a	38.92 (6.29)	0.58 (0.03)	n/a	**0.79[a] (0.04)**	1.67 (0.04)	n/a	1.74 (0.03)	1.58 (0.11)	n/a	1.72 (0.09)	6.46 (0.04)	n/a	6.42 (0.03)
	CG	15.55 (2.70)		43.88 (8.78)	0.56 (0.03)		**0.67[ab] (0.05)**	1.67 (0.03)		1.70 (0.041)	1.64 (0.09)		1.86 (0.14)	6.46 (0.03)		6.47 (0.05)
	GG	12.19 (5.09)		27.81 (15.21)	0.52 (0.05)		**0.55[b] (0.09)**	1.55 (0.06)		1.59 (0.07)	1.47 (0.16)		1.97 (0.24)	6.50 (0.05)		6.56 (0.08)
c.-3012G>C	CC	12.42 (4.56)	n/a	27.81 (15.22)	0.55 (0.04)	n/a	0.55 (0.09)	1.59 (0.06)	n/a	1.59 (0.07)	1.51 (0.14)	n/a	1.97 (0.24)	6.51 (0.05)	n/a	6.56 (0.08)
	GC	18.56 (2.54)		42.29 (8.14)	0.54 (0.02)		0.69 (0.05)	1.67 (0.03)		1.69 (0.04)	1.69 (0.08)		1.86 (0.13)	6.45 (0.03)		6.47 (0.04)
	GG	19.22 (4.14)		39.49 (6.59)	0.62 (0.04)		0.78 (0.04)	1.66 (0.05)		1.75 (0.03)	1.39 (0.13)		1.69 (0.11)	6.48 (0.04)		6.42 (0.03)

n/a – not analyzed due to low frequency of given polymorphism
